# MMPI-2-RF Profiles in Child Custody Litigants

**DOI:** 10.3389/fpsyt.2019.00725

**Published:** 2019-10-16

**Authors:** Cristina Mazza, Franco Burla, Maria Cristina Verrocchio, Daniela Marchetti, Alberto Di Domenico, Stefano Ferracuti, Paolo Roma

**Affiliations:** ^1^Department of Human Neuroscience, Sapienza University of Rome, Rome, Italy; ^2^Department of Psychological, Health, and Territorial Sciences, G. d’Annunzio University of Chieti-Pescara, Chieti, Italy

**Keywords:** Minnesota Multiphasic Personality Inventory-2-Restructured Form, custody litigants, parenting skills, personality, forensic evaluation

## Abstract

**Background and Purpose:** A psychological assessment of parents in post-divorce child custody disputes highlighted parents’ motivation to appear as adaptive and responsible caregivers. The study hypothesized that personality self-report measures completed by child custody litigants (CCLs) during a parental skills assessment would show underreporting, rendering the measures worthless. The study also analyzed gender differences in a CCL sample, general CCL profiles, and the implicit structure of the Minnesota Multiphasic Personality Inventory-2-Restructured Form (MMPI-2-RF) in the CCL sample.

**Materials and Methods:** The sample comprised 400 CCLs undergoing personality evaluation as part of a parenting skills assessment. The mean age of the 204 mothers was 41.31 years (*SD* = 6.6), with an overall range of 24–59 years. Mothers had a mean educational level of 14.48 years (*SD* = 3.2). The 196 fathers were aged 20–59 years (*M* = 42.31; *SD* = 7.8), with an average of 14.48 years (*SD* = 3.9) of education. The MMPI-2-RF was administered. To test the hypotheses, multivariate analyses of variance (MANOVAs) and two-step cluster analyses were run.

**Results:** CCL subjects reported higher scores in underreporting (L-r and K-r) and lower scores in overreporting [F-r, Fp-r, Fs-r, and response bias scale (RBS)] validity scales and restructured clinical (RC) scales, with the exception of RC2 and RC8. RC6 (Ideas of Persecution) was the most elevated. Intercorrelations within the RC scales significantly differed between CCL and normative samples. Women appeared deeply motivated to display a faking-good defensive profile, together with lower levels of cynicism and antisocial behaviors, compared to CCL men. Two-step cluster analyses identified three female CCL profiles and two male CCL profiles. Approximately 44% of the MMPI-2-RF profiles were deemed possibly underreporting and, for this reason, considered worthless.

**Discussion:** The present study adds useful insight about which instruments are effective for assessing the personality characteristics of parents undergoing a parental skills assessment in the context of a child custody dispute. The results show that almost half of the MMPI-2-RF protocols in the CCL sample were worthless due to their demonstration of an underreporting attitude. This highlights the necessity to interpret CCL profiles in light of normative data collected specifically in a forensic setting and the need for new and promising methods of mainstreaming and administering the MMPI-2-RF.

## Introduction

In any child custody evaluation, parental adequacy must be assessed in order to guarantee the best interests of the child. Among all couples who request a separation in Italy, 15–20% are subjected to psychological evaluation as part of a parental skills assessment; this percentage was released by the Supreme Court of Appeal of Rome on December 4, 2018, at the “New questions in parental competency on child custody” congress. When assessing parental fitness, examiners evaluate factors such as the social context, the child’s condition, the relationship between each parent and the child, and the personality characteristics of the child custody litigants (CCLs). Parental couples are among the more problematic in the judicial setting, as they are often in litigation over economic issues and may be less amenable to mediation agreements aimed at securing the best interests of their child. CCLs are also often characterized by impaired psychological functioning, poor coping strategies, and unrealistic ideas of themselves and others (1, 2), despite their tendency to present themselves as psychologically stable and responsible (3–7).

An overwhelming proportion of child custody evaluations involve psychometric measures, which are predominantly used to assess the personality characteristics and functioning of the litigants. One such measure, the Minnesota Multiphasic Personality Inventory-2 (MMPI-2) (8), is a well-established psychological instrument that is frequently used in forensic assessment (9–14). Ackerman and Pritzl (15), in their 20-year follow-up survey of practice and methods in child custody evaluations, highlighted that, in 97.2% of all cases, clinicians use the MMPI-2 when evaluating parents. This finding is consistent with data presented in other studies (16–18). Due to the wide use of the MMPI-2 in child custody evaluations, there is a considerable literature regarding the MMPI-2 psychometric characteristics of CCLs (4, 5, 11, 19–22, 23). This literature indicates that, overall, subjects undergoing a parental skills evaluation obtain, on some scales, significantly different scores relative to non-CCL subjects and the normative population. In more detail, CCL respondents tend to deny or omit negative features of their personality in order to present themselves in a better light, to show more adaptive psychological and behavioral functioning, and to appear as responsible caregivers who will provide for the best interests of their child. This underreporting attempt—stemming from a faking-good profile and usually combined with elevated scores on the MMPI-2 clinical scales of Hysteria (Hy), Psychopathic Deviate (Pd), and Paranoia (Pa)—is thought to be an effect of the legal environment (6, 11, 24, 25).

Recently, a restructured and shortened version of the MMPI-2, the Minnesota Multiphasic Personality Inventory-2-Restructured Form (MMPI-2-RF), was introduced (26, 27). The MMPI-2-RF is composed of items extracted from the MMPI-2 (338 vs. 567 items), arranged into 51 scales (vs. the 118 scales of the MMPI-2) (8, 28). Compared to the MMPI-2, the MMPI-2-RF has some advantages: it is shorter, it requires less time to administer, and it comprises a limited set of scales. Because it is easy to score and interpret, it reduces the potential for mistakes to be made in the assessment process, which is critically important in forensic contexts. To the best of our knowledge, only three studies have addressed the use of the MMPI-2-RF in CCL samples. In the first study, Sellbom and Bagby (7) focused on the MMPI-2-RF validity scales L-r and K-r in a group of 109 CCLs (56 men, 53 women), compared to a group of 140 university students. The students were split into two groups, with one group instructed to underreport and the other instructed to follow the standard MMPI-2 protocol. The results indicated that the CCL sample produced higher mean T-scores in the L-r and K-r scales, relative to the underreporting students; this finding underlies the role of these scales in discriminating between honest and faking-good respondents. Additionally, the authors found substantial consistency between the L-r and K-r scales, suggesting that test administrators could benefit from analyzing these scales in conjunction when making decisions about underreporting.

In the second study, Archer et al. (3) studied all MMPI-2-RF scales in a sample of 344 North American CCLs (172 men, 172 women). The authors found two major differences between this group and the general population: higher scores in underreporting validity scales (L-r and K-r) and a lower cumulative percentage frequency of restructured clinical (RC) scales with T-scores > 65. Specifically, the most commonly elevated RC scale (as shown by 15.1% of men and 18% of women) was RC6 (Ideas of Persecution). Among men, RC4 (Antisocial Behavior) was the second most commonly elevated scale, whereas RC1 (Somatic Complaints) was the second most frequently elevated scale among women. The study also examined the alpha coefficients of the H-O, Somatic/Cognitive, Internalizing, Externalizing, Interpersonal, and PSY-5 scales of the MMPI-2-RF for men, women, and the combined sample and found consistency between the internal reliabilities of these scales and those reported in the MMPI-2-RF manual. Nevertheless, the scale intercorrelation patterns were found to be very similar to those reported for other populations. The study did not examine the association between MMPI-2-RF scores and other relevant factors of individual parenting ability, and the researchers underlined that it was not possible to reach a causal inference of parents’ psychopathology on the eventual emotional disturbance of their children. Finally, Kauffman et al. (6) examined the MMPI-2-RF performance of a sample of 49 CCLs (25 men, 24 women). The results were similar to those of the previous studies of Sellbom and Bagby (7) and Archer et al. (3), indicating elevated scores in the scales of L-r (with 67% of the sample showing T-scores ≥ 55) and K-r (with 80% of the sample showing T-scores ≥ 55), in comparison to the other validity scales, which showed mean T-scores of 59.78 and 59.49, respectively. In addition, only RC6 (Ideas of Persecution) achieved a mean T-score > 50. Specifically, 43% of the sample demonstrated elevated T-scores ≥ 55, and 14% showed elevated scores in the clinical range (T-scores > 65). These results suggest that CCLs had the tendency to experiment with high levels of suspiciousness and mistrust, relative to the normative sample, and to present themselves as responsible and socially desirable.

The research of Sellbom and Bagby (7) considered only two (out of 51) MMPI-2-RF scales and acknowledged the necessity for future research to enlarge the sample for the purposes of cross validation. The results of Archer et al. (3), which considered all MMPI-2-RF scales, also require confirmation by further research. Finally, Kauffman et al. (6) findings, despite contributing to the analysis of CCL personalities, were based on a relatively small sample, which limits the generalizability of their results. Furthermore, all of the aforementioned studies administered the MMPI-2 and only retrospectively generated and scored each individual’s MMPI-2-RF, with a high risk of noisy factors (e.g., subject fatigue and overworking caused by responding to a scale of almost twice the length of the MMPI-2-RF, with item redundancy). Lastly, as reported in the literature, CCLs have specific attributes of personality and psychological functioning; thus, their MMPI-2-RF profiles should be interpreted in light of normative data collected in a forensic setting. It is also questionable whether the use of the MMPI-2-RF is altogether worthwhile, considering the different psychological characteristics of CCLs relative to the normative population (against whom data are standardized) and their common underreporting profiles, which cloud the test’s ability to discriminate by reducing values on the clinical scales. Thus, building on the research of Sellbom and Bagby (7), Archer et al. (3), and Kauffman et al. (6), the present study used the MMPI-2-RF 338-item protocol to test the following hypotheses in a large CCL sample:

H1. CCL subjects would report higher scores in underreporting validity scales (L-r and K-r) and lower scores in overreporting validity scales (F-r, Fp-r, Fs-r, and RBS), relative to the normative sample;
H2. CCL subjects would report lower scores in RC scales compared to the normative sample, and RC6 would be most elevated among CCLs;
H3. the MMPI-2-RF profiles of CCL women would differ from those of CCL men;
H4. CCL MMPI-2-RF profiles would demonstrate intercorrelations between scales that do not significantly differ from those of the normal/non-forensic population.
  Furthermore,
H5. As mean MMPI-2-RF profile scores are limited in their ability to accurately characterize individuals (because low and high scores may cancel each other out), the study tested for the presence of typical CCL personality profiles through a cluster analysis of the MMPI-2-RF scores. While this approach is not widely used in the field, it has generated important results in other settings (e.g., with respect to studies of driving under the influence of alcohol subjects and filicide).
  Finally, the study sought to investigate
H6. The percentage of underreporting MMPI-2-RF protocols in the CCL sample, expecting this to be very high.

Overall, the study aimed at testing the utility of the MMPI-2-RF in forensic settings, analyzing the percentage of useless protocols, implicit structural differences, and typical CCL profiles (in both women and men), compared to a normative sample. Given the high percentage of useless protocols due to the well-documented underreporting attitude of CCL subjects, the study was considered useful to clinicians in a position of choosing whether or not to administer this test to couples undergoing a parental skills assessment.

## Materials and Methods

### Participants

At first, the subjects were 451 parents undergoing a psychological evaluation of personality and parenting ability, as prescribed by judges in the context of a child custody dispute. Each parent agreed to participate in the study for research purposes. Thirty-six subjects compiled the MMPI-2-RF but did not give informed consent to the research, mainly because they didn’t willingly accept the CCLs assessment (consequently they refused the consent to research purpose).

In more detail, the sample comprised 196 couples plus 8 mothers whose ex-partners did not complete the MMPI-2-RF in a valid and reliable way. The 196 fathers were aged 20–59 years (*M* = 42.31; *SD* = 7.8), with an average of 14.48 (*SD* = 3.9) years of education. The 204 mothers were aged 24–59 years (*M* = 41.31; *SD* = 6.6), with a mean educational level of 14.48 years (*SD* = 3.2). No statistically significant differences were observed across genders in age and years of education, and these measures were also sufficiently aligned with the data provided for Italian divorced couples by the Italian National Institute of Statistics (29, 30). According to these latter statistics, in 2015, the majority of Italian divorced women (20.3%) were aged 40–44 years, with an average of 45 years for the entire sample; most Italian divorced men (19.7%) were aged 45–49 years, with an average of 48 years. Within this normative sample, 44.3% of women and 41% of men had a mean educational level of 13 years. The study sample was collected between 2015 and 2017 from five regional courts throughout Italy, with the collaboration of local experts in psychology who were called to evaluate parents and administer the MMPI-2-RF protocol during assessments of parental fitness. Fifteen cases were excluded, as they contained 15 or more items that were unanswered and because the Variable Response Inconsistency (VRIN-r) or True Response Inconsistency (TRIN-r) scale T-scores were ≥80. All 400 cases were court ordered, and data were only collected from child custody dispute cases; no data were collected from other child protection matters, as the literature suggests that there is a difference between these specific judicial contexts. On the one hand, child custody disputes are civil cases concerning disagreements between parents about legal and/or physical custody; on the other hand, in evaluations of parental competency, criminal charges (e.g., allegations of abuse, neglect, etc.) may co-occur, forcing the involvement of government agencies with the purpose of protecting the children involved (31).

### Materials

#### MMPI-2-RF

The full Italian version of the MMPI-2-RF (32) was used. the MMPI-2-RF (33) is a 51-scale measure of personality and psychopathology with 338 items, selected from the 567 items of the MMPI-2 (26, 34). The MMPI-2-RF has the following: nine validity scales, most of which are revised versions of MMPI-2 validity scales; nine RC scales, which were developed by Tellegen et al. and released in 2003; three higher order (HO) scales, which were derived from factor analyses to identify the basic domains of affect, thought, and behavior; 23 specific problem (SP) scales, which highlight important characteristics associated with particular RC scales; and revised versions of the personality psychopathology five (PSY-5) scales, which link the MMPI-2-RF to a five-factor model of personality pathology (26). All of the raw scores of the MMPI-2-RF scales, with the exception of the validity and interest scales, register uniform T-scores, as developed for the MMPI-2 by Tellegen and Ben-Porath (35). For these scales, a uniform T-score of 65 corresponds to the 92nd percentile and indicates the minimal level of elevation required for the interpretive recommendations. The MMPI-2-RF validity and interest scales, however, register linear T-scores, as the scales have distinct distributions, dissimilar to the composite uniform distribution. for this scale, the T-score interpretation is variable: for the TRIN-R and VRIN-R scales, T-scores > 79 could measure inconsistency; for the L scale, T-scores > 64 Could demonstrate possible underreporting; for the K scale, T-scores > 59 could show possible underreporting; and for the F “family” scales, T-scores > 79 could represent possible overreporting (relative to T-scores > 80 for Fs-R, RBS, and FBS-r).

### Statistical Analyses

To test H1 and H2, the frequency of elevation (in terms of percentile score) was studied for the seven validity scales and the nine RC scales. For the purposes of verifying H3, a multivariate analysis of variance (MANOVA) was run using gender as the independent variable and MMPI-2-RF validity and RC scale T-scores as dependent measures. The Bonferroni correction was applied for multiple comparisons. The effect sizes of the score differences between groups were recorded, with values of 0.02, 0.13, and 0.26 considered indicative of small, medium, and large effects, respectively (36). The intercorrelation for the nine RC scales in the CCL sample was compared to that of the normative sample through a z-score analysis (37), in order to verify H4. H5 was tested using a two-step cluster analysis in which the BIC criterion was used to define the profiles of female and male CCLs, respectively. This method first identified groupings using a quick cluster algorithm (pre-clustering) and then ran hierarchical cluster models in the second step. MMPI-2-RF validity and RC scales were used in the cluster model. In order to achieve natural clustering, the number of clusters was set to automatic (38). MANOVAs were also performed between gender clusters using the cluster as the independent variable and MMPI-2-RF validity and RC scale T-scores as dependent measures. Scheffé (39) method was used to assess *post hoc* pair differences (*p* < 0.05). Finally, the frequency of underreporting elevation (in terms of percentage) was also inspected for the L and K validity scales to test H6. Invalid MMPI-2-RF protocols were not included in the statistical analyses. The SPSS-18 statistical package (SPSS Inc., Chicago, IL) was used for all analyses.

## Results

### Differences Between Normative and CCL Samples

**Table 1** provides data on the frequency of elevations in the MMPI-2-RF validity and RC scales, both collapsed across genders and in the combined sample. According to the technical manual (31), in the normative sample, 10% of subjects achieved a linear T-score ≥ 65 in the validity scales, while in the RC scales, uniform T-scores of 65 fell in the 8th percentile. **Table 1** reveals that, in the underreporting scales (L-r and K-r), the percentage of CCL subjects who achieved a linear T-score ≥ 65 was almost twice the expected proportion. In the overreporting scales (F-r, Fp-r, Fs-r, FBS, and RBS), however, the percentage of CCL subjects demonstrating a linear T-score ≥ 65 was lower than the 8% expected. In relation to the RC scales, only three (out of nine) scales (RC1, RC2, and RC6) had more than 8% of CCL subjects achieving uniform T-scores ≥ 65.

**Table 1:** Frequency of elevations ≥65 for men and women on the MMPI-2-RF Validity and RC scales in the CCL sample.

	Scale	Combined (%)	Male (%)	Female (%)
**Validity scales**	L-r (Uncommon Virtues)	18.3	14.8	21.6
	K-r (Adjustment Validity)	20	16.8	23
	F-r (Infrequent Responses)	2.8	2.6	2.5
	Fp-r (Infrequent Psychopathology Responses)	2	2.6	1.5
	Fs (Infrequent Somatic Responses)	1.3	2	0.5
	FBS-r (Symptom Validity)	6	4.6	7.4
	RBS (Response Bias Scale)	2.5	2.6	2.5
**Restructured clinical scales**	RCd (Demoralization)	1.3	2.6	0
	RC1 (Somatic Complaints)	10.3	10.2	10.3
	RC2 (Low Positive Emotions)	9.8	12.2	7.4
	RC3 (Cynicism)	7.5	13.8	1.5
	RC4 (Antisocial Behavior)	5.3	8.2	2.5
	RC6 (Ideas of Persecution)	14.3	14.8	13.7
	RC7 (Dysfunctional Negative Emotions)	1.5	2	1
	RC8 (Aberrant Experiences)	2.3	2	2.5
	RC9 (Hypomanic Activation)	5.3	7.1	3.4

To evaluate whether the relationship between scales differed between the CCL and normative samples, correlation values were compared. **Table 2** shows the raw score intercorrelations between the nine RC scales, with findings for men presented in the upper diagonal and values for women presented in the lower diagonal. In the same table, the intercorrelation values reported in the Italian technical manual of the MMPI-2-RF (29) are displayed. No gender differences emerged in the correlations. Out of 36 correlations, 5 were significantly different for men, while 15 were significantly different for women. RC1 intercorrelations in both CCL women and CCL men showed the greatest differences relative to the normative intercorrelations reported in the technical manual (31). For women, most other differences were found in the RC8 scale. The great number of meaningful differences suggests that the implicit structure of the MMPI-2-RF was significantly different in the CCL sample.

**Table 2:**
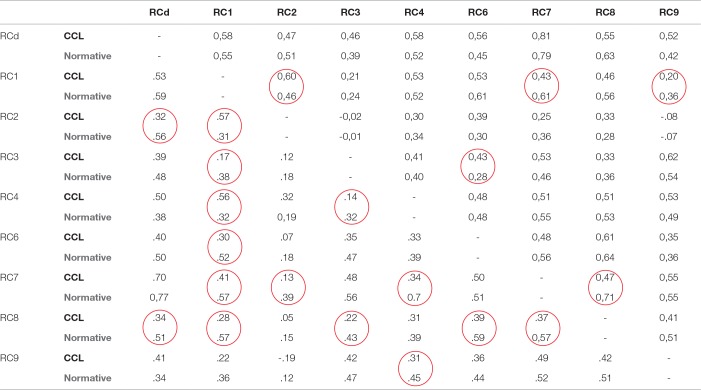
Raw score intercorrelation table for MMPI-2-RF RC scales presented separately by gender.

*Men are represented in the upper right part, women in the lower left part. CCL correlations are from the present study data; Normative correlations are from the Italian normative data. Red circles indicate differences in correlations higher than 0.15*.

In the CCL sample, approximately 44% of the MMPI-2-RF profiles could be deemed possibly underreporting and, for this reason, worthless. This estimation was based on the percentage of protocols with both linear T-scores ≥ 65 in the L-r scale and T-scores ≥ 60 in the K-r scale, in line with the cutoffs for underreporting in the technical manual (31).

### Gender Differences in the MMPI-2-RF Validity and RC Scales

A 2 × 7 MANOVA (gender × MMPI-2-RF validity scales) showed a significant gender effect on the MMPI-2-RF validity scales, *V* = 0.11, *F* (6, 393) = 8.12, p < 0.001, parη^2^ = 0.110. Separate univariate ANOVAs on the outcome variables revealed a significant gender effect on the following validity scales: L-r [F(1, 398) = 5.74, *p* = 0.017, parη^2^ = 0.014], K-r [F(1, 398) = 6.82, *p* = 0.009, parη^2^ = 0.017], and FBS [F(1, 398) = 29.38, p = 0.001, parη^2^ = 0.069].

With respect to the RC scales, a 2 × 9 MANOVA (gender × MMPI-2-RF RC scales) showed a significant overall gender effect, *V* = 0.22, *F *(9, 390) = 12.32, p < 0.001, parη^2^ = 0.221. Separate univariate ANOVAs on the outcome variables revealed a significant gender effect on the following RC scales: RC1 [F(1, 398) = 6.21, *p* = 0.013, parη^2^ = 0.015], RC3 [F(1, 398) = 35.22, p = 0.001, parη^2^ = 0.081], RC4 [F(1, 398) = 12.25, *p* = 0.001, parη^2^ = 0.030], and RC9 [F(1, 398) = 12.65, p = 0.001, parη^2^ = 0.031]. **Table 3** shows the descriptive values of the two groups (men vs. women) for all outcome variables. Compared to men, women scored higher on all significant MMPI-2-RF validity scales (L-r, K-r, and FBS) and the RC1 scale. Men had higher scores on the RC3, RC4, and RC9 scales.

**Table 3:** Mean T-scores and standard deviations for women and men for the MMPI-2-RF validity and RC scales with associated univariate F values and effect sizes.

MMPI-2-RF		Total sample *N* = 400 *M* (SD)	Women *N* = 204 *M* (SD)	Men *N* = 196 *M* (SD)	*F*	parη^2^
**Validity scales**						
	F-r	47.84 (7.08)	48.09 (6.50)	47.59 (7.66)	0.51	0.001
	Fs	45.76 (6.85)	45.97 (6.10)	45.55 (7.55)	0.37	0.001
	FBS	51.96 (8.39)	54.11 (7.90)	49.72 (8.31)	29.38^***^	0.069
	L-r	55.83 (9.15)	56.89 (8.75)	54.71 (9.44)	5.74^*^	0.014
	K-r	54.57 (8.98)	55.71 (8.26)	53.38 (9.55)	6.82^**^	0.017
	Fp-r	47.79 (6.92)	47.79 (6.66)	47.79 (7.21)	0.00	0.000
	RBS	44.84 (7.08)	45.09 (6.50)	44.59 (7.66)	0.51	0.001
**RC scales**						
	RCD	45.36 (7.60)	45.06 (6.56)	45.66 (8.56)	0.62	0.002
	RC1	49.71 (9.65)	50.88 (8.61)	48.49 (10.50)	6.21^*^	0.015
	RC2	50.27 (11.19)	50.15 (10.24)	50.40 (12.14)	0.05	0.000
	RC3	46.17 (10.29)	43.30 (7.95)	49.16 (11.55)	35.22^***^	0.081
	RC4	47.94 (8.99)	46.42 (7.78)	49.52 (9.87)	12.25^***^	0.030
	RC6	53.00 (10.48)	53.98 (10.43)	51.99 (10.47)	3.61	0.009
	RC7	44.34 (7.37)	44.02 (6.57)	44.67 (8.13)	0.77	0.002
	RC8	50.86 (6.97)	50.44 (7.13)	51.30 (6.79)	1.53	0.004
	RC9	43.75 (11.44)	41.79 (10.78)	45.80 (11.77)	12.65^***^	0.031

*p ≤ 0.05; **p ≤ 0.01; ***p ≤ 0.001.

### Cluster Analysis

The two-step cluster analysis of the 204 female CCL subjects revealed three clusters with significant differences in mean score profiles (see **Table 4**). A 3 × 16 MANOVA showed a significant clustering effect (cluster 1 vs. cluster 2 vs. cluster 3) on the MMPI-2-RF validity and RC scales, *V* = 1.21, *F *(30, 376) = 19.24, *p* < 0.001, parη2 = 0.606. In more detail, separate univariate ANOVAs on the outcome variables revealed a significant clustering effect in all MMPI-2-RF scales except for the L-r scale [*F*(2, 201) = 1.74, *p* = 0.179, parη^2^ = 0.017]. Characteristics of the CCL women in each cluster were as follows:

- Cluster 1 (*N* = 18) women had very high scores (T-scores ≥ 66) in the RC1, RC6, and RC2 scales; the FBS, F-r, RBS, F-s, Fp-r, L-r, RC8, RC4, and RC9 scales showed moderately high scores (T-scores = 55–60). All other MMPI-2-RF scales showed T-scores < 55.
- Cluster 2 (*N* = 110) women scored moderately high (T-scores > 55) to high (T-scores > 60) in the L-r scale. All other MMPI-2-RF scales showed T-scores < 55.
- Cluster 3 (*N* = 76) women scored high (T-scores ≥ 60) in the K-r scale and moderately high (T-scores = 55–60) in the L-r scale. All other MMPI-2-RF scales showed T-scores < 55.

**Table 4:** T-scores for the validity and RC scales of the MMPI-2-RF for Women-1, Women-2, and Women-3 clusters.

	MMPI-2-RF	Cluster 1 *N* = 18 *M* (SD)	Cluster 2 *N* = 110 *M* (SD)	Cluster 3 *N* = 76 M (SD)	*F*	parη^2^
**Validity scales**						
	F-r	62.94 (6.25)^a^	49.20 (3.70)^b^	42.97 (2.05)^c^	247.82^***^	.711
	Fs	59.50 (4.79)^a^	47.20 (3.70)^b^	40.97 (2.05)^c^	245.01^***^	0.709
	FBS	63.17 (8.44)^a^	54.85 (8.38)^b^	50.89 (4.46)^c^	22.56^***^	0.183
	L-r	59.39 (8.46)	55.93 (8.71)	57.70 (8.78)	1.74	0.017
	K-r	47.17 (9.36)^a^	52.95 (6.97)^b^	61.72 (5.36)^c^	55.02^***^	0.354
	Fp-r	59.50 (5.13)^a^	48.31 (5.92)^b^	44.26 (4.15)^c^	62.25^***^	0.382
	RBS	59.94 (6.25)^a^	46.20 (3.70)^b^	39.97 (2.05)^c^	247.82^***^	0.711
**RC scales**						
	RCD	53.78 (5.36)^a^	47.54 (5.08)^b^	39.42 (3.58)^c^	105.03^***^	0.511
	RC1	67.00 (8.77)^a^	52.71 (5.74)^b^	44.42 (5.10)^c^	120.59^***^	0.545
	RC2	65.83 (19.65)^a^	50.52 (7.23)^b^	45.89 (6.53)^b^	37.83^***^	0.273
	RC3	47.89 (9.87)^a^	45.01 (7.61)^a^	39.74 (6.53)^b^	15.00^***^	0.130
	RC4	56.50 (7.63)^a^	48.72 (6.91)^b^	40.70 (4.00)^c^	66.65^***^	0.399
	RC6	66.44 (10.58)^a^	57.13 (8.58)^b^	46.46 (7.27)^c^	59.19^***^	0.371
	RC7	50.50 (9.18)^a^	46.49 (4.91)^b^	38.91 (4.15)^c^	64.12^***^	0.390
	RC8	59.17 (10.22)^a^	50.10 (7.12)^b^	48.87 (4.36)^b^	18.08^***^	0.152
	RC9	56.44 (19.53)^a^	42.55 (8.64)^b^	37.22 (6.81)^c^	30.63^***^	0.234

***p ≤ 0.001. For each line, different letters indicate differences between columns.

The two-step cluster analysis of the 196 male CCL subjects revealed two clusters with significant differences in mean score profiles (see **Table 5**). A 2 × 16 MANOVA showed a significant clustering effect (cluster 1 vs. cluster 2) on the MMPI-2-RF validity and RC scales, *V* = 0.73, *F *(15, 180) = 40.97, *p* < 0.001, parη^2^ = 0.773. In more detail, separate univariate ANOVAs on the outcome variables revealed a significant clustering effect in all MMPI-2-RF scales. Characteristics of the CCL men in each cluster are summarized below. CCL men in cluster 2 scored higher in all MMPI-2-RF scales compared to CCL men in cluster 1, save for the L-r and K-r scales.

- Cluster 1 (*N* = 151) men scored moderately high (T-scores = 55–60) in the K-r and L-r validity scales. All other MMPI-2-RF scales showed T-scores < 55.
- Cluster 2 (*N* = 45) men scored high (T-scores ≥ 60) in the RC6, RC2, RC1, and RC4 scales; and moderately high (T-scores > 55) to high (T-scores > 60) in the F-r, FBS, Fs-r, Fp-r, and RBS validity scales and the RC3, RC8, RCD, and RC9 scales.

**Table 5:** T-scores for the validity and RC scales of the MMPI-2-RF for Men-1 and Men-2 clusters.

	MMPI-2-RF	Cluster 1 *N* = 151 *M* (SD)	Cluster 2 *N* = 45 *M* (SD)	*F*	parη^2^
**Validity scales**					
	F-r	44.28 (3.82)	58.69 (6.82)	330.20^***^	0.630
	Fs	42.28 (3.82)	56.53 (6.56)	335.19^***^	0.633
	FBS	47.46 (6.00)	57.31 (10.32)	64.68^***^	0.250
	L-r	55.72 (9.47)	51.36 (8.61)	7.65^**^	0.038
	K-r	56.80 (6.76)	41.91 (8.57)	147.94^***^	0.433
	Fp-r	45.38 (4.87)	55.87 (7.91)	117.30^***^	0.377
	RBS	41.28 (3.82)	55.69 (6.82)	330.20^***^	0.630
**RC scales**					
	RCD	42.44 (5.20)	56.49 (8.78)	178.44^***^	0.479
	RC1	44.60 (6.78)	61.56 (10.26)	167.77^***^	0.464
	RC2	46.84 (7.69)	62.33 (16.19)	79.15^***^	0.290
	RC3	46.32 (9,78)	58.71 (12.01)	49.91^***^	0.205
	RC4	46.13 (7.26)	60.91 (8.95)	128.65^***^	0.399
	RC6	48.72 (8.35)	62.96 (9.42)	94.98^***^	0.329
	RC7	42.01 (5.71)	53.58 (8.73)	109.07^***^	0.360
	RC8	49.34 (5.05)	57.87 (7.75)	75.51^***^	0.280
	RC9	42.76 (9.50)	56.00 (12.99)	56.243^***^	0.225

**p ≤ 0.01; ***p ≤ 0.001.

## Discussion

The main purpose of the research was to investigate if use of the MMPI-2-RF, as it is currently administered, could successfully increase our knowledge of the personality features of CCL subjects undergoing a psychological evaluation of parental fitness. The primary aim was to test the hypothesis that CCL personality profiles, as measured by the MMPI-2-RF, differ from normative profiles. This hypothesis was based on the underreporting tendencies of CCL subjects reported in the literature, characterized by elevated L-r, K-r, and RC6 scales, suggesting the motivation of these subjects to present themselves in a positive light. Furthermore, the study differentiated between CCL women and men in order to determine whether there are specific MMPI-2-RF profiles among each gender.

First, it was assumed that CCL subjects would report higher scores in the underreporting validity scales (L-r and K-r) and lower scores in the overreporting validity scales (F-r, Fp-r, Fs-r, and RBS), compared to a normative sample (H1). The results confirmed this hypothesis, in line with the aforementioned literature (3, 6, 7). CCLs showed underreporting MMPI-2-RF profiles with elevated L-r and K-r linear T-scores approximately five points higher than the medium value of the normative data. In more detail, women’s scores were almost seven points higher in the L-r scale and approximately six points higher in the K-r scale, relative to the normative sample. Men, in contrast, demonstrated an elevation of almost five points in the L-r scale and approximately three points in the K-r scale. These results aligned with the findings of Sellbom and Bagby (7), Archer et al. (3), and Kauffman et al. (6), though the latter two studies reported even higher mean T-scores for the combined sample, relative to the subjects in the present study. The CCL subjects in the present study showed elevated validity scales (L-r and K-r), with 18% demonstrating an elevated L-r scale and 20% demonstrating an elevated K-r scale at or above a T-score of 65—almost twice the 10% expected according to the standardized data. These results were especially salient for CCL women, who represented themselves as more adapted and unusually virtuous compared to normative subjects. CCL MMPI-2-RF profiles were also characterized by lower linear T-scores (ranging from two to five points) in the overreporting validity scales (F-r, Fp-r, Fs-r, and RBS), compared to the standard average. Furthermore, data on the frequencies of elevations in MMPI-2-RF validity scales reveal that such elevations should only be expected in 8% of the sample; however, a lower percentage of CCL subjects in the present study produced T-scores > 65, confirming that caregivers in child custody disputes are prone to describing themselves as more righteous, healthy, and vigorous than they effectively are. The findings with respect to the underreporting and overreporting validity scales are also consistent with other MMPI-2 research (8), which has shown CCL subjects to be more psychologically defensive than other groups, as reflected in their responses to MMPI-2 validity scales relating to defensiveness (4, 5, 19, 21, 22).

With respect to the RC scales (H2), CCL subjects in the present study scored lower than the normative sample on all but RC2 (Depressive Symptoms) and RC8 (Thinking Disorders), which showed scores in the average range. RC6 (Ideas of Persecution) was the most elevated of the RC scales, as also shown in previous studies (3, 6, 7). Elevations in the clinical range occurred most frequency in RC1 (10.3%), RC2 (9.9%), and RC6 (14.3%). Elevations above a 65 T-score in RC6 were highlighted by Kauffman et al. (6), Archer et al. (3), and Sellbom and Bagby (7). In the other RC scales, the percentage of subjects showing elevated T-scores was lower than the expected 8%, based on the normative sample. Overall, the results suggest that CCL subjects have a greater propensity to present themselves in a socially desirable way, together with higher levels of suspiciousness and mistrust and fewer displayed symptoms and feelings of negativity.

The findings support the hypothesis that there are gender differences in the MMPI-2-RF profiles of CCL subjects undergoing clinical assessment (H3), as previously highlighted by Archer et al. (3) with the MMPI-2-RF and Roma et al. (11) with the MMPI-2. In more detail, women appeared deeply motivated to display a faking-good defensive profile, together with lower levels of cynicism and antisocial behaviors, compared to CCL men. This trend could be explained by several reasons: women may have a stronger desire to gain custody of their children in order to avoid the social stigma of being judged as unsuitable mothers; mothers are generally considered the leading figures in operative caregiving, due to a rigid and conservative view of feminine roles that leads them to deny psychological imperfections; women are frequently in a weaker economical position relative to men, and this may lead them to develop a defensive attitude.

According to the fourth hypothesis (H4), it was expected that the MMPI-2-RF of the CCL sample would demonstrate a comparable implicit structure to that of a normal, non-forensic population. The findings did not bear out this assumption: rather, in contrast to the findings of Archer et al. (3), the intercorrelations reported among the nine RC scales in the CCL sample differed from those reported in the technical manual. This was true especially for women, whose RC scales showed 15 (out of 36) significantly different intercorrelations compared to women in the normative sample. This result suggests a different implicit structure of the MMPI-2-RF and highlights the need to interpret CCL profiles in the context of normative data collected specifically in a forensic setting.

In order to determine whether the MMPI-2-RF could be used to more deeply classify CCL subjects, both with and without recourse to gender, the validity and RC scales were used to define CCL typologies based on the psychological characteristics CCL subjects were aware of or wished to communicate (H5). Two-step cluster analyses showed three typical female CCL profiles and two typical male CCL profiles. Women in cluster 1 (8.8%) complained of problems related to health, cognitive symptoms, low positive emotions, and suspiciousness. In cluster 2, which comprised 53.9% of female CCLs, subjects showed a mixed profile characterized by a constricted range of feeling with limited emotional responsiveness across a wide spectrum. They also complained of medical symptomatology and unusual thoughts. Women in cluster 3 (37.3%) tended to show more adaptive psychological functioning and attempted to deny, rationalize, and limit self-disclosure, probably due to the evaluative/forensic setting. It is interesting to note that the three clusters did not differ in their communication of uncommon virtues (L-r) and thus their attitude to underreporting. Among CCL men, 77% fell in cluster 1, demonstrating underreporting profiles that masked other personality characteristics. Men in cluster 2 (23%), however, showed more problematic profiles with low positive emotion, mistrust, somatic complaints, and difficulties with people in a position of authority.

Finally, among the entire CCL sample, approximately 44% of the MMPI-2-RF profiles showed possible underreporting and, for this reason, could be considered worthless (H6). To the best of our knowledge, this was the first study to have included this kind of evaluation, digging up an overwhelming percentage of worthless protocols and calling researchers and forensic experts to join together to develop more effective methods of measuring CCL personality characteristics.

## Strengths and Limitations

One limitation of the research design is that the sample was not classified according to participant age; however, this lack of stratification was consistent with the normative group. The present study adds useful insight to the debate over the instruments that can be effectively used in forensic settings to assess the psychopathology and personality characteristics of parents undergoing a parental skills assessment. To the best of our knowledge, this study was the first to have administered the MMPI-2-RF in its own form and not to instead interpret scores that have been extracted and converted from the MMPI-2 (a similar but longer test). Moreover, the study analyzed the MMPI-2-RF protocols of men and women involved in a real forensic parenting skills evaluation, avoiding an experimental paradigm. On the basis of the results, many issues arise for researchers and practitioners. Most notably, the worthlessness of approximately half of all MMPI-2-RF protocols, due to the underreporting attitude of CCL respondents, requires the test to be administered in combination with a clinical interview and other measures (e.g., projective methods) that are less subject to simulation. This alarming finding is comparable with the results of previous studies of the MMPI-2-RF and MMPI-2 in forensic settings (40) with subjects who have driven under the influence of alcohol (13) and mothers who have committed filicide (41, 42), as well as studies on malingering (12, 14, 41). The worrying percentage of pointless protocols highlights the need to mainstream and administer the MMPI-2-RF more effectively with new and promising methods and strategies, drawing on, for instance, reaction time, machine learning, and mouse tracking (12, 43). Future studies could investigate the personality profile of CCL subjects, comparing the MMPI-2-RF with other personality assessment instruments; research could also examine whether differences exist within the personality profiles of CCLs involved in child protection matters for neglect, violence or abuse, relative to a normative population.

## Data Availability Statement

The dataset used and analyzed during the current study is available from the corresponding author upon reasonable request.

## Ethics Statement

This study was carried out with written informed consent by all subjects, in accordance with the Declaration of Helsinki. It was approved by the local ethics committee (Board of the Department of Human Neuroscience, Faculty of Medicine and Dentistry, Sapienza University of Rome).

## Author Contributions

All authors helped to conceive and plan the study and prepared and approved the final manuscript. PR conducted the data collection and produced the first draft of the final manuscript. SF, MCV, and DM supervised the data collection. PR and CM conducted the analyses and wrote the manuscript. MCV, DM, and AD carefully read the final version of the manuscript and revised it.

## Conflict of Interest

The authors declare that the research was conducted in the absence of any commercial or financial relationships that could be construed as potential conflict of interest.

## References

[1] Bonieskie LM An examination of personality characteristics of child custody litigants on the Rorschach. Diss Abstr Int (2000) 61(6-B):3271.

[2] Kennelly JJ (2002). Rorschach responding and response sets in child custody evaluations. Dissertation Abstracts International: B. The Sciences and Engineering, 63(6-B): 3034.

[3] Archer EM Hagan L Mason J Handel R Archer RP MMPI-2-RF characteristics of custody evaluation litigants. Assessment (2012) 19(1): 14–20. 10.1177/1073191110397469 21288990

[4] Bathurst K Gottfried AW Gottfried AE Normative data for the MMPI–2 in child custody litigation. Psychol Assess (1997) 9(3):205–11. 10.1037/1040-3590.9.3.205

[5] Bagby RM Nicholson RA Buis T Radovanovic H Fidler BJ Defensive responding on the MMPI-2 in family custody and access evaluations. Psychol Assess (1999) 11(1): 24–8. 10.1037/1040-3590.11.1.24

[6] Kauffman CM Stolberg R Madero J An examination of the MMPI-2-RF (Restructured Form) with the MMPI-2 and MCMI-III of child custody litigants. J Child Custody (2015) 12(2): 129–51. 10.1080/15379418.2015.1057354

[7] Sellbom M Bagby RM Validity of the MMPI-2-RF (Restructured Form) L-r and K-r scales in detecting underreporting in clinical and nonclinical samples. Psychol Assess (2008) 20(4): 370–6. 10.1037/a0012952 19086760

[8] Butcher JN Graham JR Ben-Porath YS Tellegen A Dahlstrom WG Kaemmer B MMPI-2 (Minnesota Multiphasic Personality Inventory 2): manual for administration, scoring, and interpretation, revised edition. Minneapolis, MN: University of Minnesota Press (2001). 10.1037/t15120-000

[9] Archer RP Buffington-Vollum JK Stredny RV Handel RW A survey of psychological test use patterns among forensic psychologists. J Pers Assess (2006) 87(1): 84–94. 10.1207/s15327752jpa8701_07 16856789

[10] Otto R Use of the MMPI-2 in forensic settings. J Forensic Psychol Pract (2002) 2(3): 71–92. 10.1300/J158v02n03_05

[11] Roma P Ricci F Kotzalidis GD Abbate L Lavadera AL Versace G MMPI-2 in child custody litigation: a comparison between genders. Eur J Psychol Assess (2014) 30(2):110–6. 10.1027/1015-5759/a000192

[12] Roma P Verrocchio MC Mazza C Marchetti D Burla F Cinti ME Could time detect a faking-good attitude? A study with the MMPI-2-RF. Front Psychol (2018) 9:1064. 10.3389/fpsyg.2018.01064 30090076 PMC6069678

[13] Roma P Mazza C Ferracuti G Cinti ME Ferracuti S Burla F Drinking and driving relapse: data from BAC and MMPI-2. PLoS One (2019a) 14(1):e0209116. 10.1371/journal.pone.0209116 30601844 PMC6314619

[14] Roma P Mazza C Mammarella S Mantovani B Mandarelli G Ferracuti S Faking-good behavior in self-favorable scales of the MMPI-2: a study with time pressure. Eur J Psychol Assess (2019b) 1–9. 10.1027/1015-5759/a000511

[15] Ackerman MJ Pritzl TB Child custody evaluation practices: a 20-year follow-up. Family Court Rev (2011) 49(3):618–28. 10.1111/j.1744-1617.2011.01397.x

[16] Ackerman MJ Ackerman MC Custody evaluation practices: a survey of experienced professionals (revisited). Prof Psychol: Res Pract (1997) 28(2):137–45. 10.1037/0735-7028.28.2.137

[17] Bow J Flens J Gould J MMPI-2 and MCMI-III in forensic evaluations: a survey of psychologists. J Forensic Psychol Pract (2010) 10(1) :37–52. 10.1080/15228930903173021

[18] Quinnell FA Bow JN Psychological tests used in child custody evaluations. Behav Sci Law (2001) 19(4):491–501. 10.1002/bsl.452 11568957

[19] Carr GD Moretti MM Cue BJH Evaluating parenting capacity: validity problems with the MMPI-2, PAI, CAPI, and ratings of child adjustment. Prof Psychol: Res Pract (2005) 36(2): 188–96. 10.1037/0735-7028.36.2.188

[20] Fariña F Redondo L Sejio D Novo M Arce R A meta-analytic review of the MMPI validity scales and indexed to detect defensiveness in custody evaluations. Int J Clin Health Psychol (2017) 17(2):128–38. 10.1016/j.ijchp.2017.02.002 PMC6220924 30487888

[21] Siegel JC Traditional MMPI-2 validity indicators and initial presentation in custody evaluations. Am J Forensic Psychol (1996) 14(3):55–63.

[22] Strong DR Greene RL Hoppe C Johnston T Olesen T Taxometric analysis of impression management and self-deception on the MMPI-2 in child-custody litigants. J Pers Assess (1999) 73(1):1–18. 10.1207/S15327752JPA730101 10497799

[23] Roma P Piccini E Ferracuti S Using MMPI-2 in forensic assessment. Rassegna Italiana di Criminologia (2016) 10(2): 116–122.

[24] Caldwell A Symposium conducted at the annual convention of the American Psychological Association. Interpreting MMPI data custody evaluations: a clinical perspective. In Podrygula S. (Chair), MMPI use in child custody evaluations: integrating the data (1995).

[25] Caldwell A How can the MMPI-2 help child custody examiners? J Child Custody: Res Issues and Pract (2005) 2(1/2):83–117. 10.1300/J190v02n01_06

[26] Ben-Porath YS Tellegen A Empirical correlates of the MMPI-2 restructured clinical (RC) scales in mental health, forensic and nonclinical settings: an introduction. J Pers Assess (2008) 90(2): 119–21. 10.1080/00223890701845120 18444104

[27] Tellegen A Ben-Porath YS MMPI-2-RF (Minnesota Multiphasic Personality Inventory-2-Restructured Form): technical manual. Minneapolis, MN: University of Minnesota Press (2008). 10.1037/t15121-000

[28] Butcher JN Dahlstrom WG Graham JR Tellegen A Kaemmer B, (1989). MMPI-2: manual for administration and scoring. Minneapolis: University of Minnesota Press.

[29] ISTAT Matrimoni, separazioni e divorzi. Rome: ISTAT (2015). https://www.istat.it/it/files//2016/11/matrimoni-separazioni-divorzi-2015.pdf.

[30] ISTAT Rapporto annuale 2012—La situazione del Paese. Rome: ISTAT (2014). https://www.istat.it/it/files/2012/05/Rapporto-annuale-2012.pdf.

[31] Resendes J Lecci L Comparing the MMPI-2 scale scores of parents involved in parental competency and child custody assessments. Psychol Assess (2012) 24(4):1054. 10.1037/a0028585 22612647

[32] Sirigatti S Faravelli C MMPI-2-RF. Firenze: Giunti OS (2012).

[33] Tellegen A Ben-Porath YS MMPI-2-RF (Minnesota Multiphasic Personality Inventory-2-Restructured Form): technical manual. Minneapolis, MN: University of Minnesota Press (2011).

[34] Tellegen A Ben-Porath YS McNulty JL Arbisi PA Graham JL Kaemmer B The MMPI-2 Restructured Clinical (RC) scales: development, validation and interpretation. Minneapolis, MN: University of Minnesota Press (2003).

[35] Tellegen A Ben-Porath YS The new uniform T-scores for the MMPI-2: rationale, derivation, and appraisal. Psychol Assess (1992) 4(2): 145. 10.1037/1040-3590.4.2.145

[36] Pierce CA Block RA Aguinis H Cautionary note on reporting eta-squared values from multifactor ANOVA designs. Educ Psychol Meas (2004) 64(6): 916–24. 10.1177/0013164404264848

[37] Field A Discovering statistics using IBM SPSS statistics. SAGE (2013). London: Sage Publication.

[38] Wendler T Gröttrup S Cluster analysis. In: Data mining with SPSS Modeler. Springer, Cham (2016), 587–712. 10.1007/978-3-319-28709-6_7

[39] Scheffé H The analysis of variance. New York, NY: John Wiley & Sons (1959), 351–8.

[40] Roma P Pazzelli F Pompili M Girardi P Ferracuti S (2013). Shibari: double hanging during consensual sexual asphyxia. Archi Sex Behav, (5): 895–900. 10.1007/s10508-012-0035-3 23187701

[41] Giacchetti N Roma P Pancheri C Williams R Meuti V Aceti F Personality traits in a sample of Italian filicide mothers. Rivista di Psichiatria (2019) 54(2):67–74. 10.1708/3142.31247 30985831

[42] Mazza C Monaro M Orrù G Colasanti M Ferracuti S Burla F Introducing machine learning to detect personality faking-good in a male sample: a new model based on Minnesota multiphasic personality inventory-2 restructured form scales and reaction times. Front Psychiatry (2019) 10:389. 10.3389/fpsyt.2019.00389 31275176 PMC6593269

[43] Monaro M Gamberini L Sartori G The detection of faked identity using unexpected questions and mouse dynamics. PLoS One (2017b) 12(5):e0177851. 10.1371/journal.pone.0177851 28542248 PMC5436828

